# Geochemistry and the Origin of Life: From Extraterrestrial Processes, Chemical Evolution on Earth, Fossilized Life’s Records, to Natures of the Extant Life

**DOI:** 10.3390/life8040039

**Published:** 2018-09-20

**Authors:** Satoru Nakashima, Yoko Kebukawa, Norio Kitadai, Motoko Igisu, Natsuki Matsuoka

**Affiliations:** 1 Department of Earth and Space Science, Osaka University, Toyonaka, Osaka 560-0043, Japan; 2 Undergraduate School of Physics, Osaka University, Toyonaka, Osaka 560-0043, Japan; nmatsuoka@ess.sci.osaka-u.ac.jp; 3 Faculty of Engineering, Yokohama National University, 79-5 Tokiwadai, Hodogaya-ku, Yokohama 240-8501, Japan; kebukawa@ynu.ac.jp; 4 Earth-Life Science Institute, Tokyo Institute of Technology, 2-12-1, Ookayama, Meguro-ku, Tokyo 152-8550, Japan; nkitadai@elsi.jp; 5 Department of Subsurface Geobiological Analysis and Research, Japan Agency for Marine-Earth Science and Technology (JAMSTEC), Kanagawa 237-0061, Japan; igisu@jamstec.go.jp

**Keywords:** building blocks, biopolymers, polymerization, extraterrestrial inputs, mineral surfaces, metabolism, photosynthesis, water, hydrogen bonding (9: 3-10)

## Abstract

In 2001, the first author (S.N.) led the publication of a book entitled “Geochemistry and the origin of life” in collaboration with Dr. Andre Brack aiming to figure out geo- and astro-chemical processes essential for the emergence of life. Since then, a great number of research progress has been achieved in the relevant topics from our group and others, ranging from the extraterrestrial inputs of life’s building blocks, the chemical evolution on Earth with the aid of mineral catalysts, to the fossilized records of ancient microorganisms. Here, in addition to summarizing these findings for the origin and early evolution of life, we propose a new hypothesis for the generation and co-evolution of photosynthesis with the redox and photochemical conditions on the Earth’s surface. Besides these bottom-up approaches, we introduce an experimental study on the role of water molecules in the life’s function, focusing on the transition from live, dormant, and dead states through dehydration/hydration. Further spectroscopic studies on the hydrogen bonding behaviors of water molecules in living cells will provide important clues to solve the complex nature of life.

## 1. Introduction

**Figure 1:**
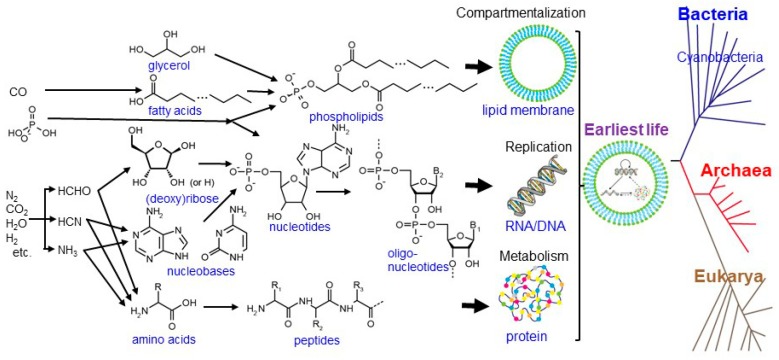
Structures, components, and abiotic synthetic pathways of bio-macromolecules operating in accordance with the three fundamental functions of life (compartmentalization, replication, and metabolism), modified from Kitadai and Maruyama [6].

Life is generally characterized by the following three functions [1]: (1) metabolism: the ability to capture energy and material resources, staying away from thermodynamic equilibrium, (2) replication: the ability to process and transmit heritable information to progeny, and (3) compartmentalization: the ability to keep its components together and distinguish itself from the environment. These functions are operated by biopolymers such as proteins, DNAs, RNAs, and phospholipids (Figure 1). Proteins are made of amino acids linked together by peptide bonds. DNAs and RNAs are made of nucleotides (composed of (deoxy)ribose and nucleobases) bound by phosphodiester linkages. Phospholipids are made of two fatty acids esterified to a glycerol phosphate molecule.

These vital components were generally assumed to be synthesized abiotically, accumulated somewhere, condensed into polymers, interacted mutually, and eventually evolved into a self-sustaining system on the primitive Earth [2,3,4,5]. A considerable number of experimental and theoretical simulations have been made to explore favorable conditions for the respective prebiotic steps. However, geo- and astro-chemical processes essential for the origin of life are still not fully clear.

To tackle these questions from Earth and planetary science perspectives, Nakashima and colleagues organized an international symposium, “Geochemistry and the origin of life,” hosted by the Interactive Research Center of Science at the Tokyo Institute of Technology on 30 March 2001. International experts in biophysics, biochemistry, geochemistry, geology, and paleontology discussed various topics leading to a publication of a book, “Geochemistry and the origin of life,” edited by Nakashima, Maruyama, Brack, and Windley [5]. This book provided our important starting points for future studies.

Here, we overview the research progress of this field since the book’s publication, with a special focus on the achievements from our group. First, we summarize the findings for the formation and survival of life’s building blocks in extraterrestrial environments, the chemical evolution of life in deep-sea hydrothermal systems, and the nature of ancient microorganisms inferred from fossilized biomolecules such as lipids. A new hypothesis on the origin and early evolution of photosynthesis is then proposed based on the changes in the redox and photochemical conditions through the Earth’s history. Besides these bottom-up approaches, the extant life’s activities with water are discussed highlighting the transition from live, dormant, and dead states for life with anhydrobiosis (life without water).

## 2. Origins of the Building Blocks of Life

### 2.1. Organics in the Cosmic Environments and Their Delivery to Earth

Since the primitive Earth around 4.6 billion years ago had abundant asteroid impacts, it is considered to have been covered with a “magma ocean”. Therefore, neither life nor its constituents could be present at least on the Earth’s surface. After the decrease of asteroid collisions, leading to the cooling of the Earth’s surface and the formation of water oceans, life is considered to have appeared around 3.8 billion years ago [7]. Most of the raw materials for life (building blocks) are then expected to have been delivered from cosmic environments, although some of them could have been synthesized inside the Earth by the processes mentioned below.

**Figure 2:**
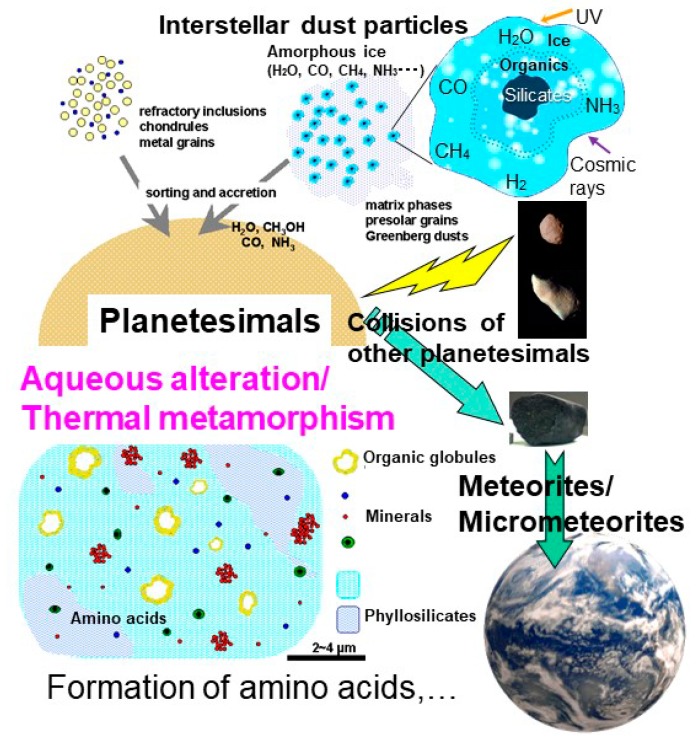
Schematic processes of evolution of organic molecules from interstellar icy dust particles, their accumulation to planetesimals, and aqueous alteration/thermal metamorphism inside the planetesimals leading to the formation of life’s building blocks such as amino acids. They could have been delivered in meteorites and micrometeorites to Earth by collisions with other planetesimals.

Organic compounds detected in cosmic environments are mainly small molecules. Some of them are considered to be adsorbed or included in an amorphous ice coating on the surface of small silicate grains (Figure 2) (e.g., [8,9]). Greenberg [9] proposed these small micrometer-sized grains as cometary dust particles that have a silicate mineral core, organic mantle, and an amorphous ice crust (“Greenberg particles”) (Figure 2), although their real structures have not yet been discovered. Ultraviolet and cosmic ray radiation are considered to induce reactions and transformations of some compounds leading to the formation of complex organic compounds including amino acids [10,11,12]. It should be noted that some submicron-sized organic nanoglobules have been observed in carbonaceous chondrites [13]. The large hydrogen isotope D/H ratios for the organic globules have been considered to indicate their pre-solar origin, at least for their raw materials [14]. They are associated with hydrous phyllosilicates but do not have silicate cores [13]. Therefore, they do not likely correspond to “Greenberg particles”.

Beyond the snow line of the protoplanetary disk, these icy dust grains accreted into planetesimals (Figure 2). The interior of some of these planetesimals might have become warm by radioactive decay heat and others (e.g., [15]). If the temperature increased, ice can be melted to liquid water containing some organic components and aqueous alteration occurred in the planetesimals’ interior (0–150 °C, for up to ≈10 million years) [16,17,18]. More heating over water evaporation could have resulted in further thermal metamorphism without water [19] (Figure 2).

To understand the nature and origins of organic matter in these interstellar or planetesimal interior environments, extensive studies on analyses of organic compounds in meteorites, micrometeorites, interplanetary dust particles (IDPs), and cometary dust particles and laboratory experiments have been conducted. Nucleic acid bases and sugar-related compounds have been detected in carbonaceous chondrites [20,21,22]. Various amino acids have also been detected from a wide variety of chondrite groups including thermally metamorphosed chondrites ([23] and references therein). Primitive chondrites with some low temperature aqueous alteration contains larger amounts of amino acids compared to thermally metamorphosed chondrites ([23] and references therein).

In order to examine the formation processes of these building blocks in extraterrestrial environments, numerous experimental studies have been conducted, in particular simulating interstellar ice chemistry and in the presence of liquid water simulating aqueous alteration in planetesimals. UV photolysis and cosmic ray bombardment of interstellar ices could produce amino acids [10,11,12], nucleobases ([24] and references therein), as well as refractory organic matter (e.g., [25]). Fischer–Tropsch type (FTT) reactions could also produce amino acids and macromolecular organic matter from high-temperature gases (CO, H_2_, and NH_3_) with the presence of mineral catalysts in the solar nebula and/or planetesimals [26,27]. In the presence of liquid water in planetesimals, amino acids could be formed by Strecker reactions from aldehydes, NH_3_, and HCN, but Strecker reactions can only produce α-amino acids (e.g., [28]). Cody et al. [29] found that complex organic solids resembling insoluble organic matter (IOM) in primitive carbonaceous chondrites were produced from formaldehyde and water starting from a formose reaction (well-known sugar formation reaction [30]) by further heating. Kebukawa et al. [31] added ammonia in this system, resulting in the formation of macromolecules with heterocyclic N and the enhancement of yields of organic solids. Kebukawa and Cody [32] determined the first order reaction rates forming these organic solids with and without ammonia. Kebukawa et al. [33] identified several amino acids in these reaction products with ammonia with relative abundances similar to those extracted from carbonaceous chondrites. These data can be considered as experimental proofs of reaction processes forming amino acids, sugars, and complex insoluble organics during aqueous alteration in planetesimals.

Some of these organics can be eventually delivered to Earth surviving radiogenic heating in planetesimals, impacts, and atmospheric entries, and used as raw materials for life after their delivery to Earth as meteorites and IDPs. Kebukawa et al. [34] studied the thermal stabilities of chondritic organics and determined decreased rates of aliphatic CHs by using in situ heating infrared micro-spectroscopy. They evaluated preservation of organics based on the above kinetic experimental data with a thermal history of an asteroid (9 km radius). For asteroids with a radius of less than 2 km, some organics were found to survive on the surface.

### 2.2. Required Conditions for Prebiotic Chemistry

Besides the extraterrestrial inputs summarized in Section 2.1, various terrestrial geochemical events have been proposed to have produced life’s building blocks as summarized by Kitadai and Maruyama [6]. Although laboratory simulations of aquatic environments on land have reported a number of great successes [35,36], demonstrated organic reactions typically start from activated carbon compounds (e.g., formaldehyde, hydrogen cyanide) that are incompatible with biologically and geologically inferred early life’s metabolism strategy [37,38,39].

Deep-sea hydrothermal systems associated with serpentinization of ultramafic rocks have been suggested to be among the most plausible settings for life to originate from both biological and geological perspectives [40]. However, experimental verification about what geochemical processes drove chemical evolution remains insufficient [41].

Recent in situ electrochemical survey of the Okinawa Trough hydrothermal fields discovered spontaneous and widespread electricity generation in deep-sea black smoker chimneys [42]. Field and laboratory investigations have verified that electrons are catalytically provided by the oxidation of reductive chemicals (e.g., H_2_S and H_2_) at the hydrothermal fluid‒mineral interface and are transported toward the outer chimney surface via electrically conductive sulfide rocks across the redox gap between the fluids and seawater [43,44,45]. Considering the ubiquity of sulfide deposits in the present-day and early ocean hydrothermal environments [46,47], together with the ever-existing redox disequilibrium between the Earth’s surface and the interior [48], the geoelectrochemical systems would have been distributed extensively on the seafloor through the Earth’s history. Kitadai et al. [49] demonstrated efficient CO_2_ electroreduction to carbon monoxide (CO) on some metal sulfides (for example, CdS and Ag_2_S) simulating early ocean hydrothermal vent environments. The reaction conditions favorable for the CO production were consistent with that assumed in Wächtershäuser’s abiotic organic synthesis starting from CO [50,51,52]. Therefore, the early ocean alkaline hydrothermal systems were suggested to have favored the prebiotic CO_2_ fixation, and for the subsequent evolution of primordial metabolism toward the origin of life [49].

The sulfide-promoted efficient CO_2_-to-CO electroreduction requires electric potentials less than −0.8 V (versus the standard hydrogen electrode; SHE) [49,53]. Occurrence of this reaction in terrestrial hydrothermal environments would be difficult because of a low boiling temperature of water (e.g., 100 °C at 1 bar) that restricts geoelectrochemically generable potential range. However, if more efficient mineral catalysts allow the electrochemical CO_2_ conversions to reactive C1 chemicals under potentials available on land (e.g., −0.7 V versus SHE in the Cedars spring systems [54]), chemical evolution starting from CO_2_ could also have been realized in terrestrial geothermal fields. In contrast to deep-sea vents, such on-land settings likely enabled the involvement of solar light as an energy source [55] and diverse organic compounds that were produced through atmospheric/photochemical reactions [56] or delivered by extraterrestrial materials [57]. In a pond near the geothermal field, water evaporation possibly led to the concentration of key precursors and their condensation into life’s building blocks [58]. Taking these advantages into account, a formamide-based volcanic origin-of-life scenario has been proposed by Saladino and coworkers [59]. It should be noted, however, that life’s origin in water-poor environments must overcome “the water paradox”: water is essential for all biological functions although its presence thwarts many proposed prebiotic reaction schemes [60]. Further experimental studies are needed to determine which of the two systems (deep-sea vs on-land) offered the best environmental situation to realize a smooth and uninterrupted transition from ancient geochemistry to biochemistry.

### 2.3. Roles of Mineral Surfaces

It has long been suggested that mineral surfaces have played a crucial role in the chemical evolution of life. The proposed functions beneficial to life’s origin include the protection, selection, and concentration of key monomers from dilute aqueous solutions [61,62,63], the activation of the adsorbed monomers’ polymerization to biopolymers [51,64,65,66,67,68,69,70,71,72,73,74], and the promotion of the emergence of biological functions such as replication, metabolism, and compartmentalization [4,75,76,77].

**Figure 3:**
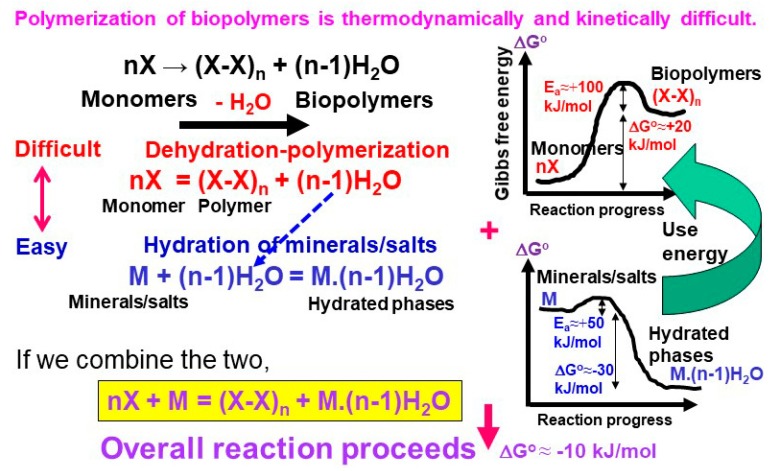
Schematic illustration of thermodynamic and kinetic difficulty for polymerization of biopolymers. Hydration of minerals/salts would enable these polymerization processes (modified from Nakashima and Shiota [79]).

Biomolecules’ polymerization in water is thermodynamically unfavorable (e.g., 17.1 kJ per one mole of peptide bond formation at 25 °C and 1 bar [78]) (Figure 3). Nakashima and Shiota [79] suggested that this difficulty can be overcome by coupling the dehydration–polymerization of monomers with the hydration of minerals when the overall reaction is thermodynamically downhill (Figure 3). This possibility was tested by Kitadai et al. [80] in a dry heating experiment of glycine with several anhydrous salts (MgSO_4_, SrCl_2_, BaCl_2_, and Li_2_SO_4_) at 140 °C for up to 20 days. They showed that glycine polymerization is promoted by the salt hydration through the hydration–dehydration interactions, and that the salt having lower hydration Gibbs energy (easier to hydrate) exhibits a greater promotion effect. This type of coupling could also occur in water on crystalline oxide minerals with low hydration ∆_r_G° (e.g., anorthite (CaAl_2_Si_2_O_3_) and forsterite (Mg_2_SiO_4_)).

**Figure 4:**
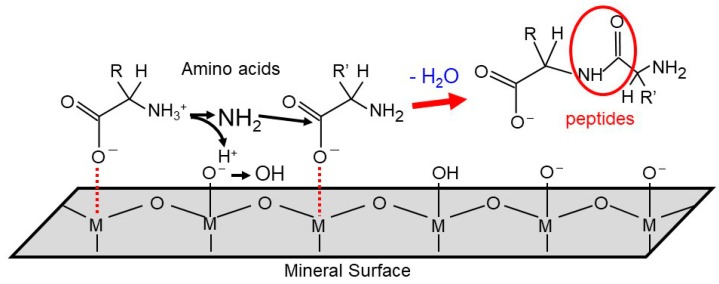
Schematic surface activation mechanisms of glycine polymerization on oxide minerals, modified from Kitadai et al. [81] with copyright permission from the journal.

Another problem regarding biomolecules’ polymerization is their sluggish reaction rates due to the presence of high activation energy barriers (Figure 3). To examine surface-catalyzed polymerization and underlying molecular mechanism, Kitadai et al. [81] compared various oxide minerals as the catalysts for glycine polymerization. Titanium oxides (rutile and anatase) were found to provide a very effective reaction site, where around 25% of glycine converted to 2- to 6-mers after 10 days’ heating at 80 °C, while only 0.01% of glycine dimerized in the absence of catalyst. Alumina (γ and α types), iron oxides (magnetite and hematite), and silica (quartz and amorphous silica) also facilitated the reaction with the degrees in this order. These observations suggest that polymerization activation arises from deprotonation of the –NH_3_^+^ group to the nucleophilic –NH_2_ one, and from the withdrawal of electron density from the carboxyl carbon to the surface metal ion (Figure 4). The surface structure and crystallinity of oxide minerals also influence the reactivity, but are not the primary factors. In addition, forsterite intermediately assisted glycine polymerization, possibly because of the hydration–dehydration interaction [81], but the basicity of the solid sample used by Kitadai et al. [81] is a more likely cause [82].

Minerals can promote biomolecules’ polymerization even in the presence of water because polymers tend to adsorb on the surface more tightly than the corresponding monomers, leading to the shift of monomer–polymer equilibria toward the polymer sides. Kitadai et al. [83] evaluated this thermodynamic effect using lysine and amorphous silica as a model combination. By combining experimentally determined adsorption constants of lysine and its dimer on amorphous silica with thermodynamic data of these organic compounds in water reported in the literature [78], it was predicted that a silica surface favors lysine dimerization, particularly at alkaline pH (pH ≈ 9) and lower ionic strength (1mM NaCl). In those conditions, around 50 times larger equilibrium dimer concentration was expected assuming infiltrating water in sandstone with a typical pore diameter (4 µm or the pore surface-to-volume ratio of 1000 m^2^ L^−1^) compared with that without silica. Importantly, the amounts of adsorption and the thermodynamically attainable dimer concentration depended greatly on the aqueous condition (e.g., pH, ionic strength). Thus, once lysine dimerization occurs in a favorable condition, changes in water chemistry can release most of the adsorbed dimers to the solution, thereby keeping the surface available for newly adsorbed species.

The thermodynamic computation as a function of environmental parameters introduced above is performable only for the lysine–amorphous silica system because of a lack of adsorption constants for polymers. However, the surface affinities for ion adsorption of oxide minerals are theoretically predictable [84,85]. Amino acid adsorptions on oxide minerals are controlled mostly by electrostatic interaction [86,87]. Therefore, future experimental characterizations of some representative acidic, basic, and neutral amino acids (e.g., lysine, aspartate, and alanine) and their corresponding peptides by the methodology reported by Kitadai et al. [83] will enable evaluation of favorable geological settings for peptide bond formation on the primitive Earth.

Recently, amorphous silica was found to stabilize ribose [88] and promote its condensation with phosphate and adenine into adenosine monophosphate under a dry condition [89]. Nucleotides might also be synthetized in hydrothermal conditions [90].

## 3. Origin and Evolution of Photosynthesis: A New Hypothesis

The first life that appeared on Earth might have used metabolic pathways presented above in sea floor hydrothermal environments like present day hyperthermophiles. They might have lived in sulfide-rich hydrothermal vent environments with a primordial metabolic system that preceded the autotrophic CO_2_ fixation such as the reductive acetyl-CoA pathway and the reductive tricarboxylic acid (rTCA) cycle [91,92].

With the evolution of Earth’s interior forming a strong magnetic core, Earth is supposed to have become surrounded by a magnetic field shielding cosmic electromagnetic radiation around 3 billion years ago [7]. This might have enabled ancient microbes to survive at shallow sea environments. The microbes then started to use solar energy for metabolism which started photosynthesis.

**Figure 5:**
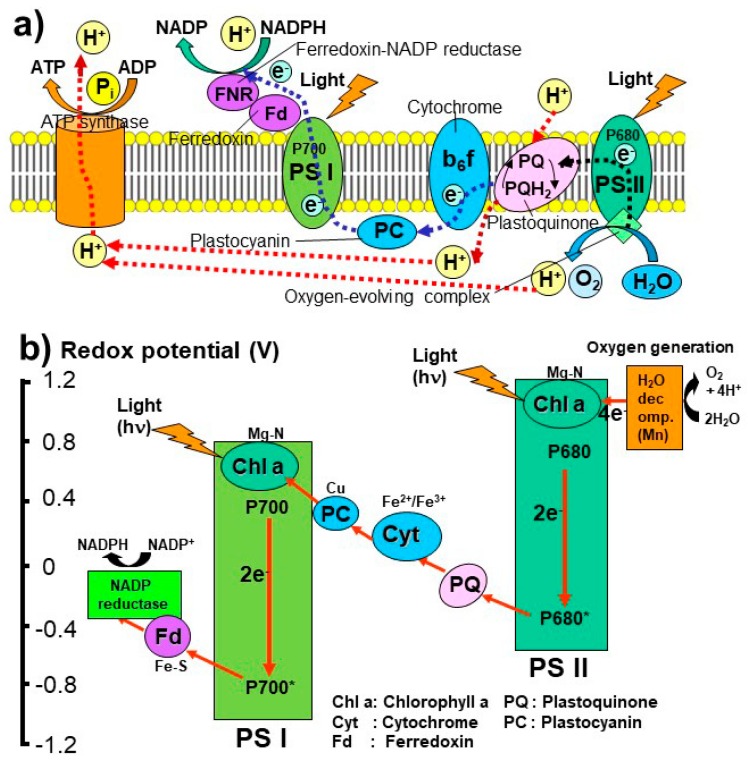
(**a**) Present day photosynthetic pathways in the lipid membrane and (**b**) redox potentials of the photosynthetic systems I (PSI and II (PSII), modified from Blankenship [95] and Martin et al. [96].

The present-day photosynthesis operates with complex electron and proton transfer pathways by using many photosynthetic pigments, such as chlorophylls absorbing lights at 680 nm (P680) and at 700 nm (P700), and proteins such as cytochrome and ferredoxin to produce nicotinamide adenine dinucleotide phosphate (NADP) and adenosine triphosphate (ATP) (Figure 5) [93,94,95,96]. Recent genomic comparison of different photosynthetic microbes suggests that the photosynthetic systems I (PS I) and II (PS II) occurred separately and merged into oxygen generating systems in cyanobacteria and plants by means of endosymbiosis [95,96]. 

**Figure 6:**
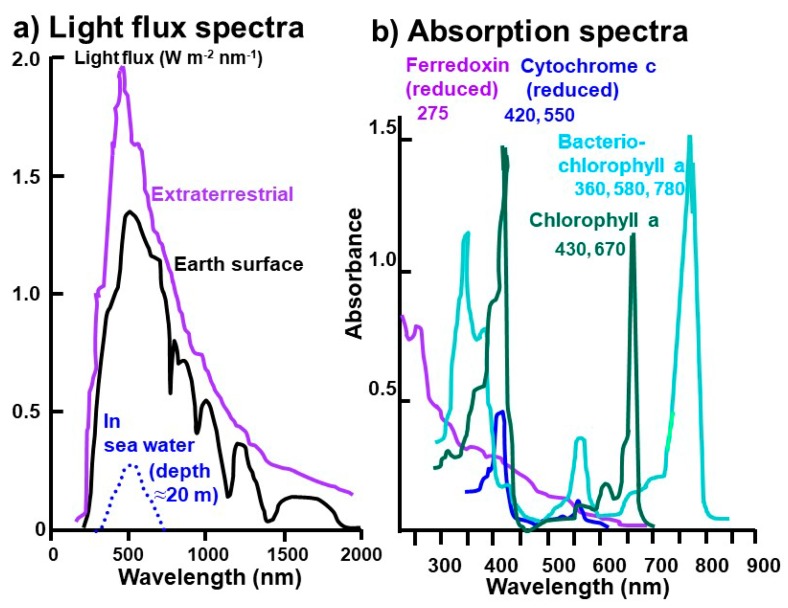
(**a**) Present day light flux spectra from the Sun, modified from Blankenship [95], Smith et al. [97], and Seinfeld and Pandis [98]. (**b**) Absorption spectra of photosynthetic pigments and proteins, modified from Blankenship [95] and Wang et al. [99]. Copyright permission was obtained from the journal, the book, and the author.

Sun lights arriving at the present Earth’s surface is mostly in the 300 to 1300 nm wavelength range, while those at the depth of 20 m in sea water is narrower around 500 nm (Figure 6a) [95,97,98]. On the other hand, major photosynthetic pigments do not use lights around 500 nm but absorb 275 nm for ferredoxin, 420 and 550 nm for cytochrome, 430 and 670 nm for chlorophyll a, and 360, 580, and 780 nm for bacteriochlorophyll a (Figure 6b) [95,99].

Spectral characters of sunlight arriving at the Earth’s surface and sea water around 3 billion years ago are not known but could be different from the present-day ones due to different compositions of the ancient atmosphere and ocean. Earth’s atmosphere without oxygen (absorbing UV <240 nm) and ozone (absorbing UV <360 nm) [98] will permit UV light to reach Earth’s surface and sea water.

Under these UV-rich oxygen-free environments around 3 billion years ago, the first photosynthetic molecules might have been absorbing UV light under reducing conditions. Therefore, ferredoxins with Fe-S centers with the lowest redox potential could have been the first photosynthetic molecule absorbing UV light around 275 nm (“proto-PS I”) (Figure 5 and Figure 6). This Fe-S containing protein could have been formed during the above “proto-metabolism” operating on the surface of metal sulfides.

With the further evolution of Earth’s atmospheric and oceanic environments, wavelengths of sunlight might have shifted from UV to the visible region and the redox potential might have also increased. Cytochrome with Fe^2+^/Fe^3+^ pairs could have been the second photosynthetic molecule (“proto-PS II”), absorbing light around 420 and 550 nm (Figure 5 and Figure 6).

Chlorophylls might have been added later to these proto-PS I and II systems with further changes in atmospheric and oceanic conditions. They can work as antenna for absorbing mainly 680 and 700 nm in high (oxidizing) redox conditions leading to oxygen generating systems in cyanobacteria around 2.7 billion years ago.

This new hypothesis on the origin and evolution of photosynthesis proposed here based on geochemical redox and light environments is just a working one without mechanistic details. Several biologists [100,101,102,103,104] have proposed that the primitive photosynthetic reaction centers were porphyrin—FeS couples (PSI type) or quinone types [95]. Kritsky et al. pointed out that excited flavins, which can photocatalyze reactions leading to the accumulation of free energy in the products, were available in the earliest stage of evolution [105]. Recent genomic and molecular structural comparison studies on various photosynthetic microbes suggested that at least either PS I or PS II systems might have started separately and then merged into oxygen generating systems in cyanobacteria and plants by means of endosymbiosis [95,96]. Because it is extremely difficult to test this hypothesis by detecting remnant photosynthetic molecules in the fossil record, this hypothesis cannot be confirmed easily. However, a new view based on geochemical environmental evolution of Earth should be considered in the origin and evolution of photosynthesis. In particular, ultraviolet-visible spectra arriving at the Earth’s surface and sea water should be evaluated quantitatively by assuming atmospheric and oceanic compositions. We are currently working on the formation and degradation pathways and rates of photosynthetic pigments in present day plants in order to find clues for their evolutional pathways.

## 4. Fossilized Evidence of Life

The remnant of life in Archean (about 4–2.5 billion years ago) rocks has the potential for elucidating the origin of life on Earth. The presence of life on Earth has been inferred mainly from morphologically preserved microscopic fossils, stromatolites, molecular biomarker, and stable isotopic compositions in sedimentary rocks. Recently, the oldest putative microfossils were reported from ≈3.8 billion-year-old ferruginous sedimentary rocks in Quebec, Canada [106]. In addition, the ^13^C-depleted graphite was found from ≈3.95 billion-year-old metasedimentary rocks in Labrador, Canada, which provides the oldest evidence of life on Earth [38]. However, because of scarcity and the poor preservation of Archean rocks, interpretation of the ancient records for the presence of life is still controversial.

As an example, cell-like structures found in ≈3.5 billion-year-old Apex chert (sedimentary rock composed of microcrystalline quartz) in Australia have been taken as the oldest microfossils [107]. However, Brasier et al. [108] questioned the biological origin of these structures and suggested that they can be formed by precipitation of organics from hydrothermal solutions. On the other hand, the chemical analyses of isolated organic matter have provided important information about the physiological and phylogenetic characteristics of the source organic compounds (e.g., [109,110]). However, the bulk analysis of isolated organic matter generally involves the potential risk of post-depositional and experimental contamination of the organic matter (e.g., [110,111]).

In situ analysis have provided isotopic, elemental, and molecular signatures of individual microstructures in petrographic thin sections (e.g., [112,113,114,115,116,117,118,119,120,121,122,123,124,125,126,127,128,129]). Among these chemical signatures, molecular characteristics of microfossils have been obtained using spectroscopic techniques including Raman micro-spectroscopy, infrared (IR) spectroscopy, electron energy-loss spectroscopy (EELS), and X-ray absorption near-edge structure (XANES) spectroscopy. Raman micro-spectroscopy has been used to identify mineralogy of the samples. Especially for evaluating microfossils, Raman micro-spectroscopy has been used to clarify their carbonaceous composition; that is G (graphite) and D (disordered or defect) bands [114,115,116,117,118,124,125,128,130,131,132]. However, it should be noted that these G and D bands can also be observed for experimentally produced abiotic organic matter, and thus are not necessarily indicators for their biogenic origin [133]. These bands are also often used as an indicator for evaluating the maturation degree of carbonaceous matter in sedimentary rocks (e.g., [134,135]).

**Figure 7:**
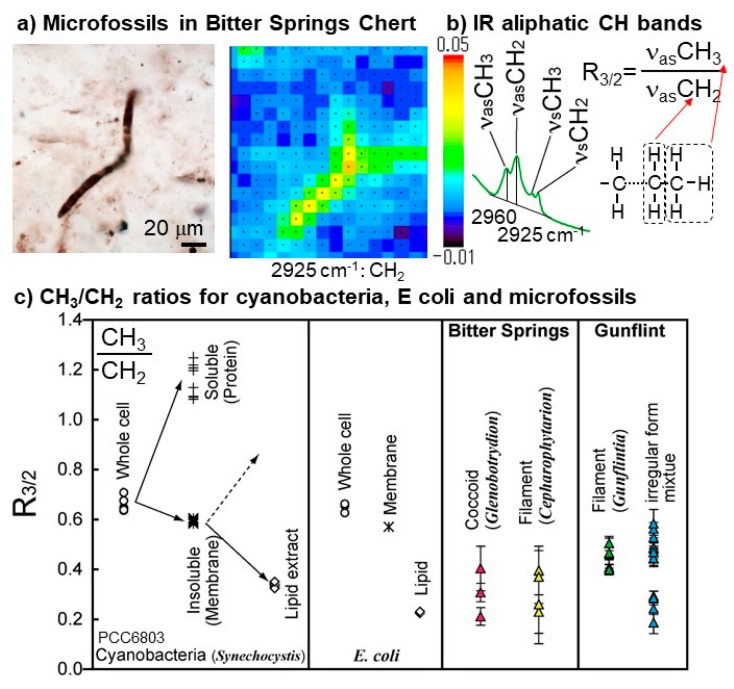
Infrared (IR) micro-spectroscopic analyses of microfossils. (**a**) Optical microscope image of a filamentous microfossil in Bitter Springs chert and IR map image for 2925 cm^−1^ peak height due to aliphatic CH_2_ modified from Igisu et al. [120]. (**b**) Schematic images for determining the CH_3_/CH_2_ peak height ratio (R_3/2_). (**c**) CH_3_/CH_2_ peak height ratio (R_3/2_) of extant prokaryotes (cyanobacteria and E coli), and microfossils from Bitter Springs Group and Gunflint Formation modified from Igisu et al. [121] with copyright permission from the journal. Values for the whole cell, water-soluble (proteins), water-insoluble (membrane), and lipid extracts are also shown for cyanobacteria and *E. coli*.

IR micro-spectroscopy revealed distributions of aliphatic C–H bonds corresponding to microfossil structures in Bitter Springs (0.83 billion year old) [120] and Gunflint cherts (1.9 billion year old) [121] (Figure 7a). The CH_3_/CH_2_ peak height ratios of microfossils, which correspond to relative length and/or branching of aliphatic chains of lipids [136], were found to be similar to those of modern cyanobacteria [121] (Figure 7b,c). Aliphatic C–H bonds have also been detected in microfossils and/or carbonaceous matter in Wumishan stromatolites (≈1.5 billion years) [137] and Doushantuo phosphorites and cherts (≈0.58 billion years) [137,138].

EELS combined with transmission electron microscope (TEM) showed that organic matter from Apex chert possesses C=C of amorphous carbon [139]. XANES spectroscopy was applied to Apex and Gunflint carbonaceous matter [140] and Gunflint microfossils [141]. They revealed the presence of COOH and aromatic COH components in Apex and Gunflint organic matter [140], and the presence of amide derived from protein in Gunflint microfossils [141].

These in situ methods detecting molecular information would be useful for identification and/or classification of microfossils. Although the functional groups such as C–H, C=C, COOH, and COH can be included in non-biogenic carbonaceous compounds, such as asphalt [60], their spatial distribution data by the in situ methods corresponding to microfossil structures would support their biogenicity. However, post-depositional degradation of microorganisms should be considered for comparison of the obtained data with extant microorganisms. Some experimental studies were performed in order to evaluate changes in the spectroscopic signatures during fossilization process [121,141,142]. It is important for a better understanding of the post-depositional degradation of microorganisms to accumulate information on the durability of organic components under various preservation conditions.

## 5. Roles of Water for Life through Studies on Anhydrobiosis

The presence of liquid water is essential for life’s activities with functions of biomolecules. However, some microbes, plants, and insects can survive in the absence of water and this tolerance against anhydrous states is called “anhydrobiosis” (life without water) [143]. This anhydrobiosis can keep a sort of sleeping state (dormancy) during dehydrated states and can recover viable states upon rehydration. Recent biological studies suggested the following two important mechanisms for anhydrobiosis [143,144,145]: (1) protection of proteins against denaturation by means of surrounding sugars such as trehalose, and (2) fixation of cellular structures by changing lipid membranes into glassy states. Therefore, studies on transitions from live, dormant, and dead states through dehydration/hydration might provide clues for life’s functions with water.

Several studies indicated usefulness of infrared (IR) spectroscopy for characterizing molecular changes of lipids and proteins [144,146]. However, IR spectroscopic signatures of transitions from dormancy to live states and from live states to dormancy or death have not been elucidated.

**Figure 8:**
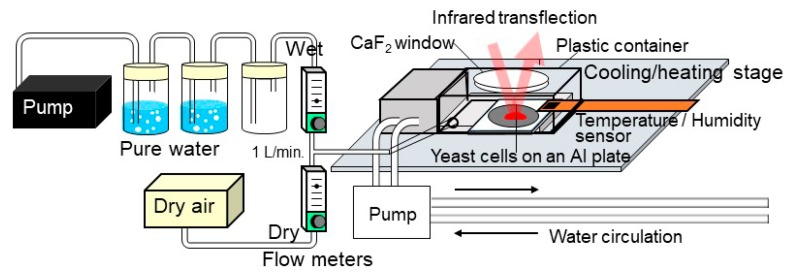
In situ IR micro-spectroscopy system with controls of relative humidity (RH) and temperature (T) for studying changes of yeast cells during dehydration/rehydration and/or heating/cooling.

Yeast (*Saccharomyces cerevisiae*) is one of the unicellular eukaryotic microbes employed in fabrication of breads. Dried yeast cells are known to be in dormancy and can return back to activities upon rehydration. We are now studying these transition processes among dormancy, viable states, and death by means of an original in situ IR microspectroscopy system with controls of relative humidity (RH) and temperature (T) (Figure 8). RH in a plastic container with a CaF_2_ window was controlled by flowing a mixture of dry and wet air. By changing the flow rates of the dry and wet air with flowmeters to be the total flow rate at 1 L min^−1^, the mixed air introduced to the plastic cell can be controlled from about 2 to 80% RH [147]. Dry yeast powders were placed on an Al plate on a Peltier cooling–heating stage (−20 to 120 °C) in the RH/T control cell (Figure 8). IR transflection (transmission–reflection) spectra were measured every 1 minute during RH/T changes.

**Figure 9:**
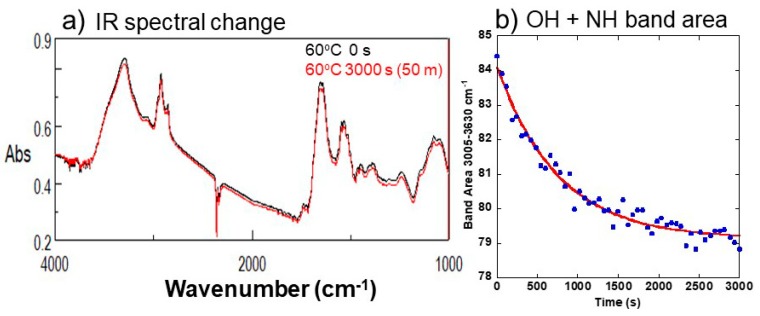
(**a**) IR spectral changes with time of the yeast cells heated at 60 °C for 0 and 3000 s. (**b**) Changes with time for the first 3000 s in the IR band area at 3005–3630 cm^−1^ (OH + NH) of the yeast cells heated at 60 °C. A fitting exponential curve is also shown.

Aggregated dry yeast cells dispersed with one drop of pure water placed on an Al plate showed the following IR absorption bands (Figure 9a): a broad band at 3700–3000 cm^−1^ due to OH and NH stretching of water molecules and proteins (amides A and B); a group of several bands at 3000–2770 cm^−1^ due to CH stretching of lipids; two bands at 1720–1480 cm^−1^ due to C=O stretching (amide I), and CNH (amide II) and HOH bending of proteins and water molecules; and a broad band at 1170–1000 cm^−1^ due to C–O stretching of sugars.

Our preliminary heating experiments of the yeast cells at 60 °C at ambient room RH condition showed IR spectral changes. In the first stage (0 to 3000 s), OH+NH (Figure 9b), amides I+II+H_2_O, and CH band areas decreased exponentially. In the later second stage, OH+NH and amides I+II+H_2_O band areas increased gradually, while the CH band area continued to decrease gradually. Changes with time for the first 3000 s in band areas at 3005–3630 cm^−1^ (OH + NH)(Figure 9b), at 1480–1720 cm^−1^ (amides I + II + H_2_O), and at 2775–3005 cm^−1^ (aliphatic CH) could be fitted by exponential equations to determine the first order reaction rate constant k_1_ to be 1.3 × 10^−3^ s^−1^ for the OH + NH band area, 0.93 × 10^−3^ s^−1^ for the amides I + II + H_2_O band area, and 0.87 × 10^−3^ s^−1^ for the aliphatic CH band area. These reaction rate constants are all on the order of 1 × 10^−3^ s^−1^, showing similar decay constants. Since the 3005–3630 cm^−1^ band area corresponds to stretching vibrations of OH in water and NH in proteins, and that at 1480–1720 cm^−1^ to peptide bonds in proteins (amides I, II) with a contribution of bending vibration of H_2_O molecules, these band area decreases indicate a loss of water associated with protein changes.

Since yeast cells are considered to lose viability at 60 °C, the above decreases in water, proteins, and lipids can be corresponding to a loss of life’s functions possibly due to a loss of hydrogen bonding. Although further detailed studies are required, the present first results indicate the usefulness of the new method for elucidating transitions among dormancy, live, and dead states, leading to a better understanding of the life’s function with water.

The life’s functions are mostly maintained by hydrogen bonding: (1) secondary and higher order structures of proteins are formed by hydrogen bonding mainly among peptide bonds, (2) information transfer is operated by base pairing among DNAs and RNAs by hydrogen bonding, (3) lipid membranes separating cellular compartments have polar head groups facing to liquid water by hydrogen bonding, and (4) liquid water molecules containing all these biopolymers are hydrogen bonded to each other. Therefore, hydrogen bonds are considered to be essential for life’s activities [148] and further studies on the nature of hydrogen bonds among and within biopolymers and water molecules are necessary.

## 6. Summary and Future Perspectives

(1) Extraterrestrial inputs

Primitive meteorites contain a wide variety of organic compounds, including potential building blocks of life, such as amino acids, nucleobases, and sugar-related compounds. These organic compounds would have been formed and evolved in various environments, e.g., icy dust in molecular clouds and planetesimals. Some of these would have been delivered to the early Earth within meteorites, comets, and IDPs. Despite the numerous works on meteorite analyses and experimental studies, the origins and evolutions of organic compounds are not fully understood. Future sample return missions such as Hayabusa2 and OSIRIS-REx would provide more clues for origin, evolution, distribution, and delivery to Earth of prebiotic molecules in space environments.

(2) Prebiotic chemistry

Laboratory experiments have demonstrated the syntheses of almost all of life’s building blocks and their polymers under simulated prebiotic conditions. However, how these compounds spontaneously assembled to generate proto-biological functions (e.g., metabolism, replication) on the Hadean Earth remains unclear. Geochemically plausible CO_2_ fixation systems and their developments in response to the acquisition or synthesis of simple organic molecules are the next important topics to be explored for constraining environments and mechanisms for life to originate on Earth and other Earth-type planets.

(3) First metabolism and photosynthesis

The “proto-metabolism” might have initiated in the geoelectrochemical environments in deep-sea alkaline hydrothermal systems through abiotic CO_2_ reduction and fixation into carboxylic acids (e.g., TCA intermediates) on metal sulfides.

The photosynthesis then started in shallow sea water. Although the origin and evolution of complex photosynthetic systems are still under debate among biologists, geochemical environments, such as redox and light conditions, should be considered. The first photosynthetic molecule could have been ferredoxins with Fe-S centers, which could have been formed by the above proto-metabolism, absorbing UV light around 275 nm (“proto-PS I”) under UV-rich oxygen-free Earth environments around 3 billion years ago. Cytochrome with Fe^2+^/Fe^3+^ pairs could have been the second photosynthetic molecule (“proto-PS II”), absorbing light around 420 and 550 nm. Chlorophylls absorbing mainly 680 and 700 nm at high (oxidizing) redox conditions could have been added later to these proto-PS I and II systems, leading to oxygen generating systems in cyanobacteria around 2.7 billion years ago.

(4) Fossil records

Geological records provide direct evidence of the presence of life on Earth in four main ways: microfossils, stromatolites, molecular biomarkers, and stable isotope ratios. However, interpretation of the ancient records of the presence of life is still under debate because Archean rocks are rare on Earth, and poorly preserved. In situ analyses of individual microstructures reveal their elemental, isotopic, and molecular compositions, and enable us to examine spatial relationships between individual morphological structures and the surrounding minerals. Further careful descriptions of such microscopic chemical and morphological information are necessary for better understanding of their biogenicity.

(5) Water, hydrogen bonds, and life

Studies on transitions from live, dormant, and dead states through dehydration/hydration might provide clues for life’s functions with water. Our preliminary heating experiments of the yeast cells at 60 °C at an ambient room RH condition by using an original in situ IR microspectroscopy system with controls of relative humidity (RH) and temperature (T) showed decreases in water, proteins, and lipids. They can correspond to a loss of life’s functions, possibly due to a loss of hydrogen bonding. Further studies on the nature of hydrogen bonds among and within biopolymers and water molecules are necessary.

Since the publication of the book entitled “Geochemistry and the origin of life” in 2001, the first author (S.N.) tried with colleagues to conduct the following four research targets: (1) material proofs of building blocks of life, mainly from extraterrestrial materials; (2) experimental evidence of formation processes of building blocks and polymers, both in extraterrestrial and terrestrial environments; (3) roles of mineral surfaces on the chemical evolution; and (4) molecular signatures of ancient life’s fossil records. These studies could constrain some favorable environments for origin and evolution of life to be (a) aqueous alteration in planetesimals, (b) seafloor hydrothermal environments for polymerization and proto-metabolism, and (c) some mineral surfaces. Although most of the analytical and experimental studies in the literature showed inventories and showcases of building blocks and polymers, our group has provided thermodynamic and kinetic constraints with some logical background on the chemical evolution to life.

These studies suggest that mineral–water–organic interfaces are often playing key roles in the origin and evolution of life. Therefore, further interdisciplinary researches linking cosmochemistry, geochemistry, mineralogy, organic chemistry, electrochemistry, and photochemistry to investigate quantitatively mineral–water–organic interactions are required. They are not only important in the origin and evolution of life in the past, but also in quantitative prediction of Earth’s resources and environments toward our future.
